# Skeletal Muscle Mass as a Potential Mediator of the Physical Activity–Left Atrial Function Relationship: A Community-Based Cross-Sectional Study

**DOI:** 10.3390/jcm15197744

**Published:** 2026-10-08

**Authors:** Yue Zhong, Kai Li, Lijun Zeng, Xue Yang, Changwei Cai, Bin Yu, Shujuan Yang, Qingyu Dou

**Affiliations:** 1 Department of Cardiology, West China Hospital, Sichuan University, Chengdu 610041, China; 2 Department of General Internal Medicine, West China Second Hospital, Sichuan University, Chengdu 610041, China; 3 National Clinical Research Center for Geriatrics, Center of Gerontology and Geriatrics, West China Hospital, Sichuan University, Chengdu 610041, China; 4 Outpatient Department, West China Tianfu Hospital, Sichuan University, Chengdu 621203, China; 5 The Clinical Hospital of Chengdu Brain Science Institute, MOE Key Lab for Neuroinformation, University of Electronic Science and Technology of China, Chengdu 610036, China; 6 West China School of Public Health and West China Fourth Hospital, Sichuan University, Chengdu 610041, China

**Keywords:** physical activity, left atrial strain, skeletal muscle mass index, cardiac function, mediating effect

## Abstract

**Objectives:** Left atrial (LA) strain reflects early cardiac dysfunction preceding structural remodeling and clinical disease. Data on the association between physical activity (PA) and LA function are limited. In this study, we aim to explore the association of PA with LA strain, and to test the indirect effect of skeletal muscle mass in the PA-LA association. **Methods:** Data from the Healthy Lifestyle Promotion Cohort involving 407 participants were analyzed. Multivariate linear regression models were used to estimate the associations of leisure-time PA with LA function, and indirect associations through skeletal muscle mass were determined using a regression-based mediation analysis. **Results:** PA was significantly associated with LA reservoir strain (β = 2.907, *p* < 0.001) and with pump strain (β = −1.674, *p* < 0.001). PA had a significant effect on the skeletal muscle mass index (SMI) (β = 0.230, *p* < 0.001), while the SMI had a significant impact on LA reservoir strain (β = 2.619, 95% CI 1.425–3.813, *p* < 0.001) and pump strain (β = −2.621, 95% CI −3.472–−1.771, *p* < 0.001). The SMI partially mediated the effect of PA on LA reservoir strain by 21% (95% CI: 9–38%, *p* < 0.001) and LA pump strain by 36% (95% CI: 20–59%, *p* < 0.001). Subgroup analysis revealed that this statistical mediation effect of the SMI demonstrated a stronger trend in women compared to men and in participants ≥60 years old compared to their younger counterparts. **Conclusions:** The SMI partially mediates the association between PA and LA function in the community-dwelling Chinese participants. Sustaining a higher level of leisure-time PA was associated with better LA function. These possible beneficial effects may be partially explained through favorable changes in the SMI.

## 1. Introduction

Left atrial (LA) function, including parameters like LA volume index, LA ejection fraction, and speckle-tracking echocardiography-derived LA strain, has emerged as a sensitive predictor of cardiovascular outcomes and cognitive impairment, such as atrial fibrillation, heart failure, stroke and dementia [1,2,3]. Impaired LA strain reflects subclinical dysfunction preceding structural remodeling and clinical disease [4,5]. The comprehensive assessment of LA dysfunction is clinically valuable, providing key information for early cardiovascular risk evaluation. Physical activity (PA) is a well-established modifiable lifestyle factor strongly associated with reduced cardiovascular morbidity and mortality [6]. While the cardioprotective effects of PA are recognized, evidence regarding the benefit of PA on LA function in community-dwelling people remains limited. The ARIC (Atherosclerosis Risk In Communities) study observed that higher leisure-time PA was associated with larger LA size but better LA function [7]; however, the biological pathways mediating the benefits of PA on LA function remain incompletely elucidated.

In the context of global aging, skeletal muscle mass has garnered significant attention, and it may serve as a key mediator linking PA to LA function. PA influences skeletal muscle mass by directly stimulating muscle anabolism, thereby helping to preserve or increase muscle protein synthesis [8]. Beyond its mechanical role, skeletal muscle is recognized as an important endocrine and metabolic regulator, influencing systemic inflammation, insulin sensitivity, and various neurohormonal pathways, many of which are further implicated in cardiac health [9]. The loss of skeletal muscle mass and function, termed sarcopenia, is associated with worse outcomes in cardiovascular disease [10]. The study by Bekfani et al. reveals a significant correlation between muscle function and left atrial function in heart failure patients [11], supporting that skeletal muscle may play a mediating role in the PA-LA function relationship. Further investigation into this association may provide novel insights into cardiovascular disease prevention and management.

Specifically, previous studies have typically treated muscle mass as either a covariate or an independent predictor, rather than testing the indirect effect through which leisure-time PA may influence cardiac function. Our study therefore aims to fill this gap by formally testing whether skeletal muscle mass serves as a statistical mediator in the relationship between leisure-time PA and LA strain in a community-dwelling population. Further, it was feasible to evaluate the index of skeletal muscle mass in a large community-based sample through body composition analysis. Other potential mediators such as cardiorespiratory fitness (which requires exercise testing) and inflammatory markers (which require additional blood assays) were not measured, although these factors may also contribute to the PA–LA relationship, because our study was designed to test the specific hypothesis that skeletal muscle mass mediates this association, rather than to compare multiple competing pathways. We specifically tested the indirect effect of physical activity on LA strain through indices of skeletal muscle mass (including skeletal muscle mass index). Focusing on a community-based cohort allows for the examination of graded variations in PA and muscle mass in a population largely free of overt cardiovascular disease, which is ideal for identifying early, subclinical pathways and developing personalized prevention thresholds applicable to asymptomatic adults. Understanding this potential role of skeletal muscle may offer insights into the pathway linking PA to LA function and could inform future strategies aimed at preserving cardiovascular health through musculoskeletal assessment.

## 2. Method

### 2.1. Study Population and Ethics

This study utilized data from the Healthy Lifestyle Promotion Cohort (HLPC), which employed a multistage random sampling design to recruit local residents from four counties in Sichuan Province, China. The baseline survey was conducted in 2018, with follow-up assessments in 2020 and 2023. The detailed characteristics and establishment of the HLPC have been described previously [12]. For the present cross-sectional analysis, we focused on participants who completed the 2023 survey, which included concurrent assessments of PA, body composition, and echocardiography. Inclusion criteria for the current study were: (i) being aged between 30 and 79 years old; (ii) not being injured or physically limited; and (iii) having complete data on assessments of PA, body composition, and echocardiography. Participants were excluded if they had atrial fibrillation, congenital heart disease, significant valvular heart disease, or left ventricular ejection fraction <55%, or if their echocardiographic image quality was insufficient for reliable speckle-tracking analysis. Participants with missing data on PA, body composition, and echocardiography were not included in the analysis; thus, no imputation methods were applied. Every participant provided written informed consent. This study was approved by the Sichuan University Medical Ethical Review Board (K2016038) and the Ethics Review Committee of West China Tianfu Hospital, Sichuan University (2022[006]), and conducted in accordance with approved guidelines. The reporting of this cross-sectional study follows the STROBE (Strengthening the Reporting of Observational Studies in Epidemiology) Statement guidelines for cross-sectional studies.

### 2.2. Clinical Evaluation

All participants underwent a standardized protocol comprising a face-to-face interview, physical examination, laboratory tests, body composition analysis, muscle function measurements, and echocardiography. Personal disease history, medication history and smoking status were recorded. Blood pressure was averaged from two measurements with 1 min intervals after 5 min seated rest. Blood samples were tested for triglyceride, low-density lipoprotein cholesterol, serum creatinine and hemoglobin A1c levels.

### 2.3. Leisure-Time PA

Leisure-time PA over the preceding three months was assessed using the validated International Physical Activity Questionnaire (IPAQ)-long form [13]. The Chinese version of the IPAQ has been previously validated in multiple Chinese populations, demonstrating acceptable reliability and validity [14,15]. The IPAQ-long form assesses PA across four domains: occupational, transportation, household, and leisure-time. For the present study, we focused exclusively on the leisure-time PA domain. For specific leisure-time activities, we focused on the following categories: Tai Chi/Qigong/walking, brisk walking/dancing, running, swimming, ball sports, apparatus-based exercises, and mountain climbing/other. Then PA was weighted by estimated energy expenditure using the metabolic equivalent of task (MET). The MET levels for specific PA were determined using the 2024 Adult Compendium of PA for adults aged 19–59 years and the Older Adults Compendium of PA for individuals aged 60 years and older [16,17]. This approach utilizes the most current and age-appropriate MET values for retrospective calculation. These compendia served as coding references rather than measurement instruments so that the later publication date would not introduce temporal bias. The weighted MET minutes per week were calculated by multiplying the duration by the frequency per week and the MET intensity. The level of overall leisure-time PA was classified into five categories: 0 (totally sedentary), <500, 500–999, 1000–1499, and ≥1500 MET-min/week.

### 2.4. Body Composition and Muscle Strength Measurements

Body composition was assessed using a multi-frequency bioelectrical impedance analyzer (InBody 770 analyzer, Seoul, Korea). This device estimates lean mass and fat mass in different body parts. Fat-free mass was derived as the difference between body weight and fat mass, and then the fat-free mass index (FFMI) was obtained after normalization by the square of body height. The skeletal muscle mass index (SMI) was calculated by dividing the sum of the muscle masses of the four limbs by height squared. The site-specific SMI was represented by the upper-limb SMI (USMI) and lower-limb SMI (LSMI), computed by dividing the corresponding muscle mass by height squared. We conducted these assessments at a consistent time of day, after an overnight fast, and with participants avoiding vigorous exercise prior to measurement. The Jamar Hydraulic Digital Hand Dynamometer (Sammons Preston, Bolingbrook, Illinois, USA) was used to assess hand grip strength, which was quantified in kilograms, and the highest value of the two readings for the dominant hand was selected for the subsequent analysis.

### 2.5. Cardiac Structure and Function

Echocardiography was performed by two blinded medical doctors using standard equipment (Philips, EPIQ7C, Bothell, WA, USA). Imaging data were analyzed off-line after being saved in digital format. Cardiac dimensions were measured according to recommendations of the American Society of Echocardiography and the European Association of Cardiovascular Imaging [18]. Left ventricular (LV) mass was calculated by the modified cube formula. The early peak (E) was assessed from the apical 4-chamber view. Pulsed-wave tissue Doppler was used to evaluate peak early diastolic tissue velocity (e’) at the septal sides of the mitral annulus. LV end-diastolic and end-systolic volumes in the apical four-chamber and two-chamber views were used to calculate the LV ejection fraction (LVEF). LV global longitudinal strain (LVGLS) was analyzed as the average of the measurements in two-chamber, three-chamber, and four-chamber views. LA emptying fraction was calculated as (maximal LA volume − minimal LA volume)/maximal LA volume × 100%. The assessment of LA deformation was performed by semiautomated 2-dimensional speckle tracking (Qlab version 16, Philips systems, Bothell, WA, USA), analyzing all segments of LA in the apical four- and two-chamber views. The onset of the QRS complex was used as the reference point (R-R gating). Deformation curves were automatically generated and displayed, and the software computed average strain for all LA segments from both apical views. The LA reservoir strain was defined as the peak LA strain value during the cardiac cycle. LA pump strain (corresponding to atrial contractile function) was measured as the value of strain at the onset of the P-wave in the electrocardiogram, and LA conduit strain was determined as the difference between LA reservoir strain and pump strain. The reported median value of normal LA reservoir strain ranged from 30% to 50% [19,20,21]. Accordingly, we divided the cohort of participants into two groups based on a threshold of 40% (mean value of the cohort = 43.6%). This cutoff was cohort-mean-derived and used for descriptive comparison only (not applied in the primary regression or mediation models, which treated LA strain as a continuous variable). All echocardiographic parameters were averaged over 3 consecutive cardiac cycles.

### 2.6. Statistical Analysis

All statistical analyses were performed using R software (version 4.2.0). Categorical variables were described by frequencies (percentages) and continuous variables by means (standard deviation, SD). Group differences based on LA reservoir strain status were compared using Student’s *t*-test or the chi-square test, as appropriate. Bivariate analyses served as a preliminary screening step to identify which skeletal muscle indices (e.g., SMI, FFMI, USMI, LSMI) and which left atrial function parameters (e.g., LA reservoir, conduit, pump strain) were statistically associated with PA and therefore warranted inclusion in the subsequent regression-based mediation analyses. For multivariate linear regression analyses, PA was entered as a continuous ordinal variable to test for a linear trend across PA categories. Covariates for multivariable adjustment were pre-specified a priori based on clinical knowledge. Model 1 was adjusted for age, sex, LVEF, and comorbidities (hypertension, diabetes, coronary heart disease). Model 2 was further adjusted for LVGLS. Model 3 additionally included the use of antihypertensive and antidiabetic medications. To examine the statistically mediating role of skeletal muscle, we first assessed the association between PA and muscle indices (FFMI, SMI, USMI, LSMI, grip strength), and then estimated the effect of PA on LA function when skeletal muscle indices were controlled. The regression-based mediation approach proposed by Imai et al. was further used to examine the indirect effects of muscle indices in the associations between PA and LA function [22]. In this approach, the total effect could be decomposed into direct effects (not mediated by muscle mass and function) and indirect mediation effects (mediated by muscle mass and function). The percentage of the indirect mediation effect over the total effect was calculated for each mediator. Subgroup analysis was performed to explore modification effects of sex and age in the associations between PA and LA function. In particular, menopausal status was included as an adjustment variable in the female subgroup. To determine effect sizes and obtain 95% confidence intervals, we assessed direct, indirect, and total effects using the bootstrap method with 1000 iterations, which is consistent with methodological recommendations for stable percentile confidence intervals [23]. A significant effect was shown when the 95% confidence interval did not include zero. A two-sided *p*-value < 0.05 was considered statistically significant. Multiple comparisons were corrected using the Benjamini–Hochberg false discovery rate (FDR) method, and adjusted Q < 0.05 was considered statistically significant.

## 3. Results

### 3.1. Sample Characteristics

The analytical sample comprised 407 participants from the HLPC who had complete data on leisure-time PA, body composition, and echocardiography (Table 1). Participants with atrial fibrillation (*n* = 5), significant valvular heart disease (*n* = 8), left ventricular ejection fraction <55% (*n* = 9), congenital heart disease (*n* = 1), or inadequate image quality (*n* = 6) were excluded. The selection process of the study participants is summarized in a flowchart (Supplementary Figure S1). The study population had a mean age of 56.61 ± 11.59 years and included 177 males and 230 females. Significant differences in age, body mass index, and the prevalence of coronary heart disease were observed when participants were stratified by an LA reservoir strain threshold of 40% (the mean value of the cohort was 43.6%, and the established normal range is 30–50%). Higher systolic blood pressure was associated with lower LA reservoir strain (137.3 ± 18.9 mmHg for LA reservoir strain <40% vs. 132.7 ± 19.8 mmHg for those with ≥40%, *p* = 0.02). LA and LV strain indices, the LA volume index, leisure-time PA, the FFMI, the SMI, the site-specific SMI and grip strength also differed significantly between the two groups. Pearson correlation analysis indicated significant mutual correlations between PA level, skeletal muscle mass indices, and LA reservoir strain (all *p* < 0.05; Supplementary Figure S2).

### 3.2. Associations Between PA and LA Function

**Figure 1:**
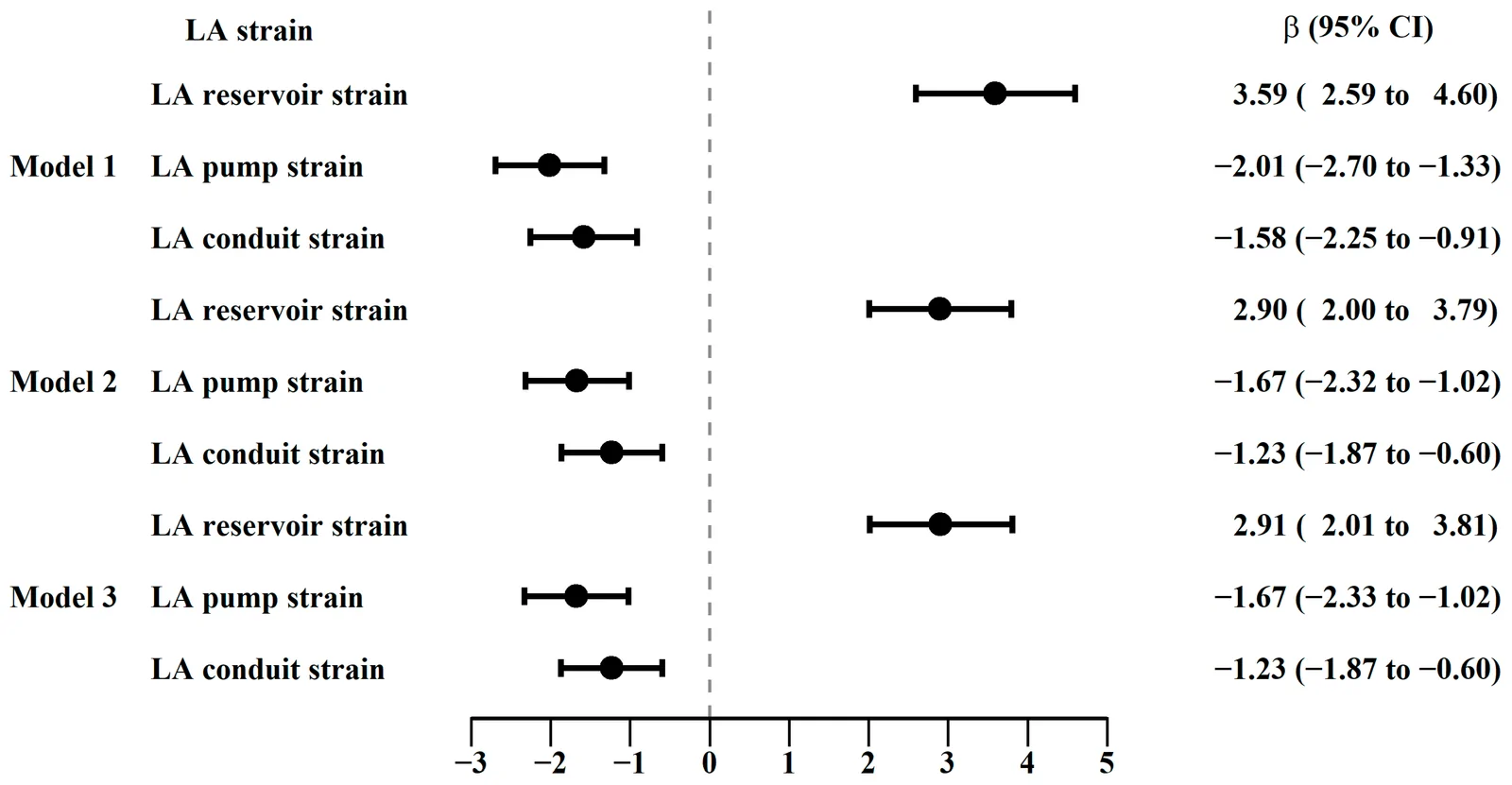
The association between physical activity and left atrial strain. Model 1 included adjustments for age, sex, left ventricular ejection fraction, and the comorbidities hypertension, diabetes and coronary heart disease. Model 2 added left ventricular longitudinal strain, and Model 3 further added the use of antihypertensive drugs and antidiabetic drugs. LA, left atrial.

In the total effects regression (Figure 1), a relatively higher level of PA was a significant predictor of better LA function, and this association remained significant after sequential adjustments in multivariate regression analyses (Figure 1). In the fully adjusted Model 3, PA was significantly associated with all three LA strain parameters. Each one-category increment in PA level was associated with a 2.91% change in LA reservoir strain (β = 2.91, *p* < 0.001), a 1.23% change in conduit strain (β = −1.23, *p* < 0.001), and a 1.67% change in pump strain (β = −1.67, *p* < 0.001). The direction of these associations, which is positive for reservoir strain and negative for conduit and pump strains, reflects the distinct biomechanical actions of the LA during different phases of the cardiac cycle. Subgroup analyses revealed consistent patterns in both male and female participants, as well as in those under 60 years of age (Supplementary Table S1). However, among participants aged 60 years or older, PA did not exhibit a significant association with LA conduit function.

### 3.3. Statistical Mediation Effects of Skeletal Muscle Mass

**Figure 2:**
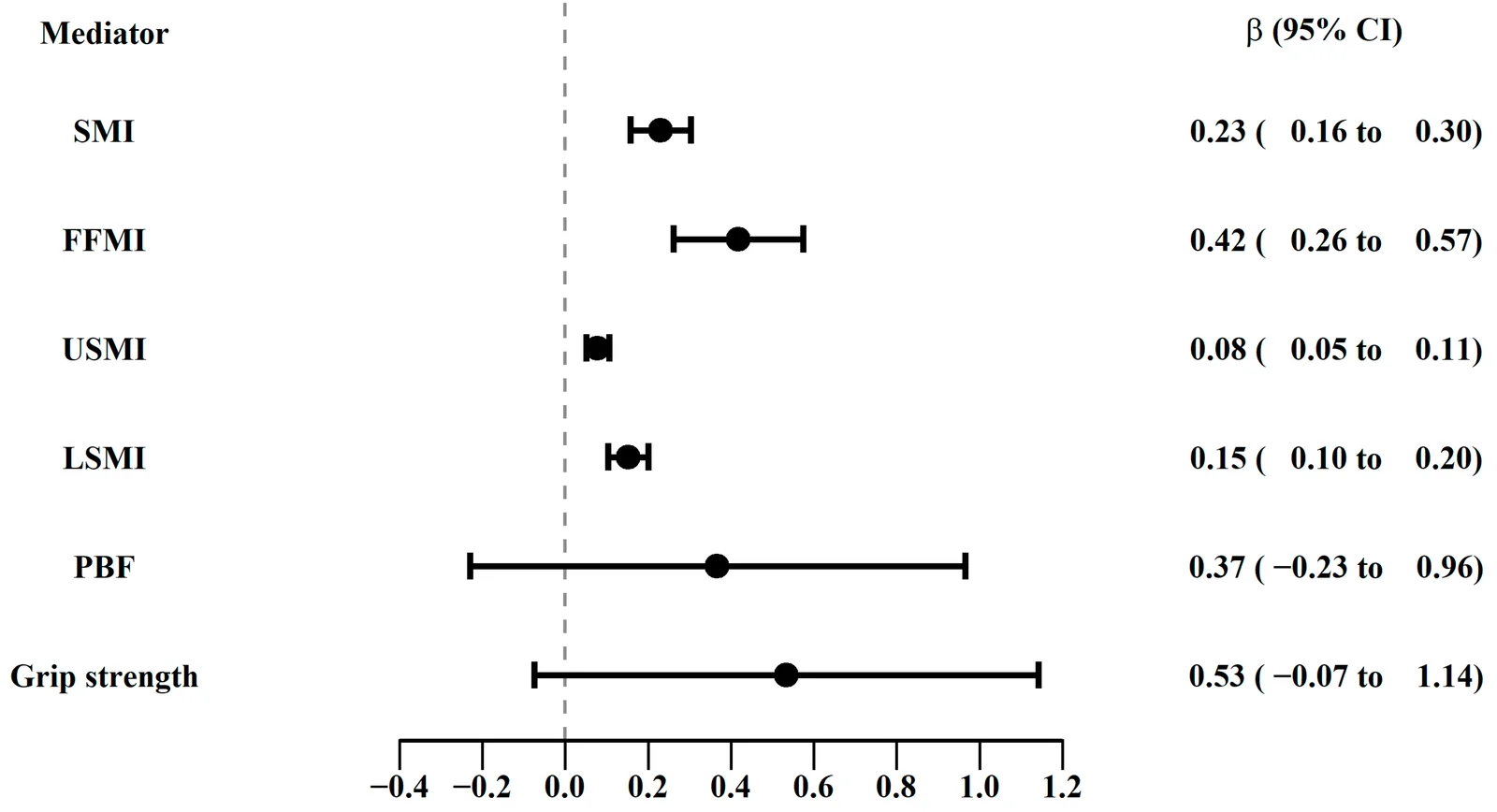
The association between physical activity and indices of skeletal muscle. SMI, skeletal muscle mass index; FFMI, fat-free mass index; USMI, skeletal muscle index of upper limbs; LSMI, skeletal muscle index of lower limbs; PBF, percent body fat.

**Figure 3:**
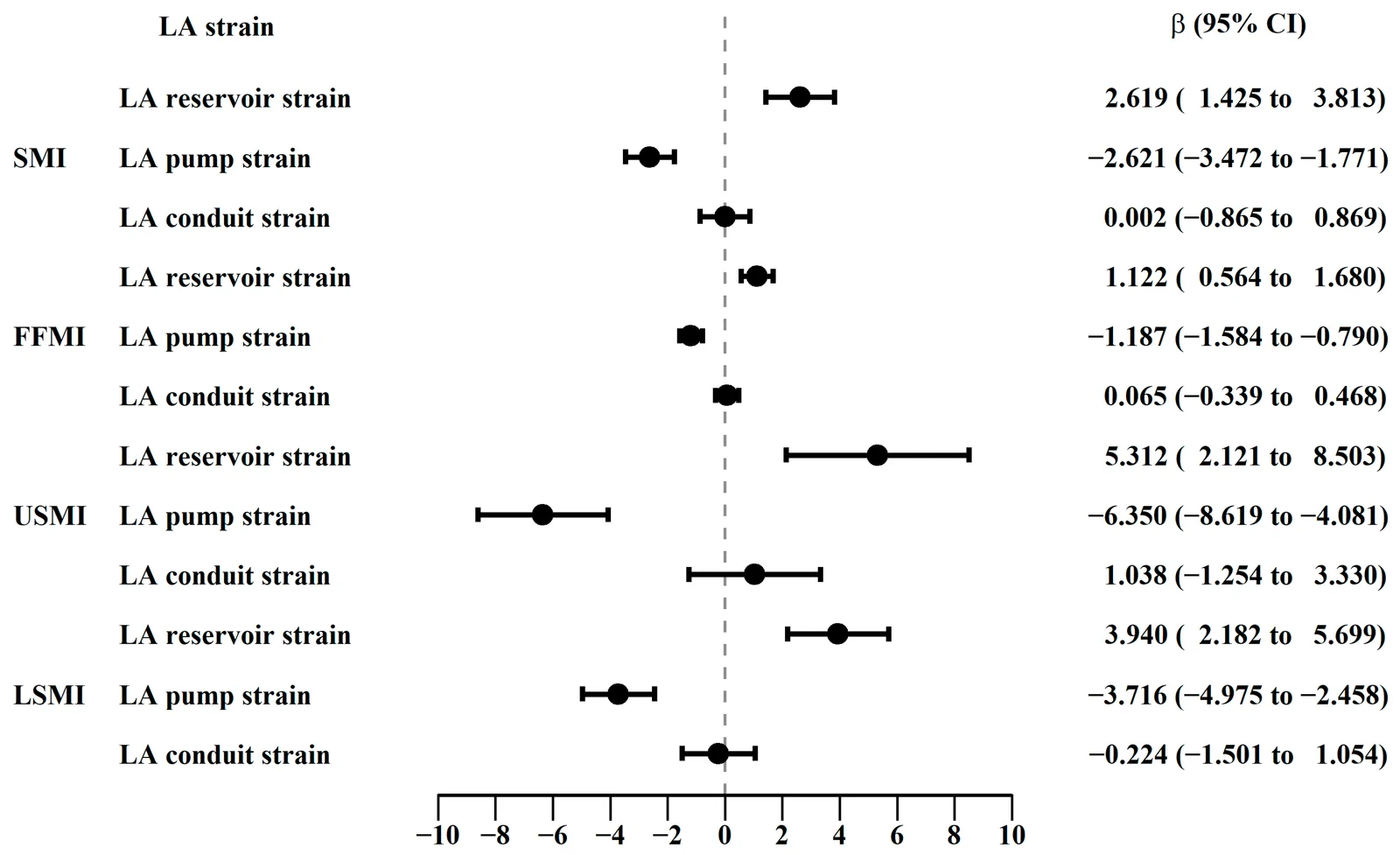
The association between indices of skeletal muscle mass and left atrial strain. LA, left atrial; SMI, skeletal muscle mass index; FFMI, fat-free mass index; USMI, skeletal muscle index of upper limbs; SMI, skeletal muscle index of lower limbs.

**Figure 4:**
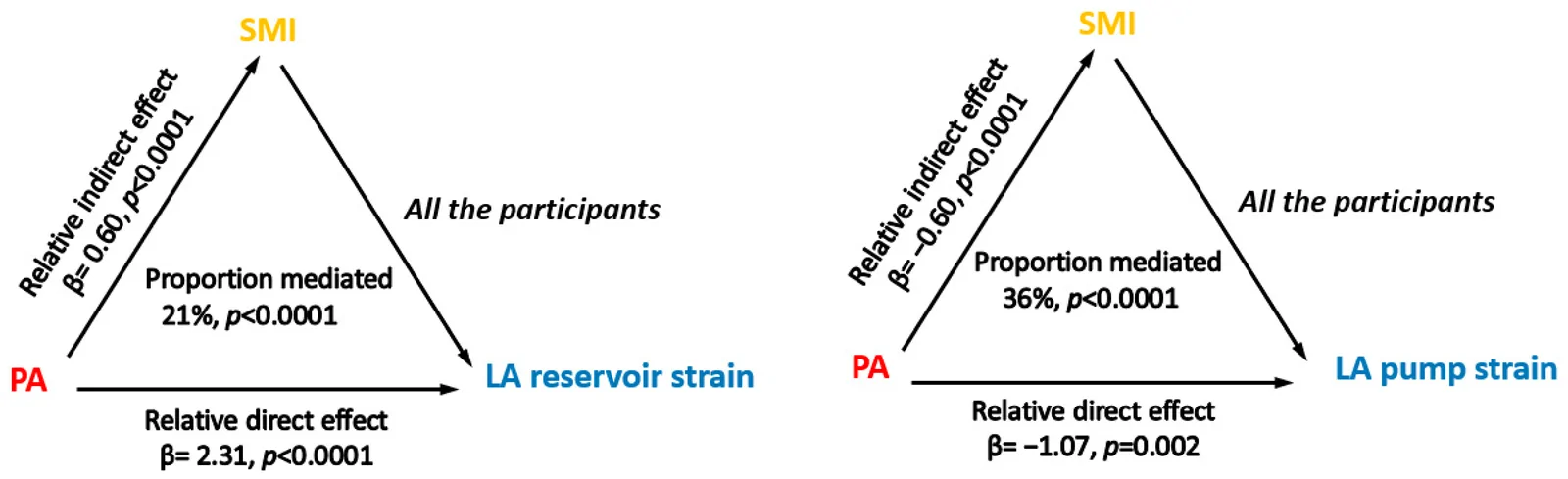
Statistical mediation effect of the SMI in the associations between PA and LA reservoir strain and between PA and LA pump strain. LA, left atrial; PA, physical activity; SMI, skeletal muscle mass index.

As illustrated in Figure 2, leisure-time PA was significantly correlated with all indices of skeletal muscle mass (including the SMI, USMI, LSMI, and FFMI). The correlations between indices of skeletal muscle mass and LA function are presented in Figure 3. Higher SMIs, USMIs, LSMIs, and FFMIs were significantly associated with greater LA reservoir strain and higher absolute values of LA pump strain. No significant correlation was observed between any skeletal muscle index and LA conduit strain. Therefore, conduit strain was not included in the mediation analysis. Mediation analysis using the bootstrap method revealed a significant indirect effect of PA (indirect effect = 0.60, 95% CI: 0.27 to 1.04, *p* < 0.001, Q < 0.05) on LA reservoir strain via the SMI (Table 2 and Figure 4), accounting for 21% (95% CI: 9% to 38%, *p* < 0.001) of the total effect. For LA pump strain, the mediated effect through the SMI was also significant (indirect effect = −0.60, 95% CI: −0.86 to −0.36, *p* < 0.001, Q < 0.05), representing 36% (95% CI: 20% to 59%, *p* < 0.001) of the total effect (Table 2 and Figure 4). The statistical mediation effects of the FFMI, USMI and LSMI are shown in Supplementary Table S2. The mediation proportions for the FFMI were 16% (95% CI: 6% to 33%, *p* < 0.001) for reservoir strain and 30% (95% CI: 16% to 54%, *p* < 0.001) for pump strain. The LSMI demonstrated a stronger mediating trend than the USMI for both LA reservoir strain (21% vs. 14%) and pump strain (34% vs. 30%).

Subgroup analyses further indicated that the statistical mediating effect of the SMI varied by sex and age. For LA reservoir strain, the SMI statistically mediated 13% of the association in males (indirect effect = 0.37, 95% CI: 0.02–0.94; *p* = 0.04) and 25% in females (indirect effect = 0.77, 95% CI: 0.31–1.31; *p* < 0.001). By age, the mediation proportions were 17% in participants under 60 years (indirect effect = 0.49, 95% CI: 0.13–0.91; *p* = 0.006) and 33% in those aged 60 or older (indirect effect = 0.92, 95% CI: 0.33–1.71; *p* < 0.001). A numerically larger mediation effect was consistently observed for LA pump strain than for reservoir strain across all subgroups (Table 2). However, it should be noted that subgroup analysis was exploratory and was not corrected by multiple comparisons. The subgroup analysis for the indirect effects of the FFMI, USMI and LSMI is shown in Supplementary Table S2. The statistical mediating effects of these skeletal muscle indices were found in the <60-year-old, ≥60-year-old, and female subgroups. Among male participants, however, the FFMI and USMI had no indirect effects in the association between leisure-time PA and LA reservoir strain. Additionally, in female participants, the mediating effect of the LSMI was numerically larger than that of the USMI for both LA reservoir and pump strain, whereas this pattern was not evident in males (Supplementary Table S2).

## 4. Discussion

In this community-based cross-sectional study, higher levels of leisure-time PA were positively associated with favorable LA strain. More importantly, statistical mediation analyses indicated that appendicular skeletal muscle mass, mainly quantified by the SMI, partially explained this association. Similar patterns were observed across sex and age subgroups, underscoring the potential role of skeletal muscle mass as a mediator in the relationship between habitual PA and LA function.

To date, limited studies have revealed the relationships between leisure-time PA and LA function in the general population. The ARIC study, conducted among community-dwelling older adults, indicated that higher leisure-time PA was associated with improved LA reservoir strain [7]. However, this benefit may not extend to high-intensity exercise regimens. Extreme and persistent endurance exercise can induce atrial fibrosis and inflammation, which might cause atrial fibrillation [24]. The Tromsø Study, 1994–2016, reported that moderate PA lowered the risk of atrial fibrillation in both men and women and in all age groups, even in people with LA enlargement [25]. In our research, higher leisure-time PA was related to better LA strain in the whole cardiac cycle. It is worth emphasizing that only a small proportion of participants engaged in high-intensity PA (5.7% exceeding 1500 MET-min/week and only 2.2% exceeding 3000 MET-min/week). Because the present cohort included few participants with very high PA levels, and PA was modeled as ordinal categories, we could not reliably assess potential nonlinear (e.g., U- or J-shaped) associations. Future studies with a wider distribution of PA and continuous exposure modeling are needed to examine this possibility.

Skeletal muscle mass may statistically account for part of the association between PA and cardiac health, suggesting a potential pathway that warrants further investigation. As a major metabolic organ, skeletal muscle can affect glucose disposal and lipid oxidation [26]. Additionally, skeletal muscle pump function enhances venous return and modulates peripheral vascular resistance [27], thereby potentially benefiting LA function. Our data indicate that the SMI partially mediated the relationships of PA with both LA reservoir strain and LA pump strain, with a numerically stronger mediating effect on LA pump strain compared to reservoir strain. This finding may be explained by the differential susceptibility of atrial functional components to age-related changes. LA reservoir strain is often impaired in the early stages of cardiac disease, whereas LA pump strain may exhibit compensatory enhancement before progressing to a decompensated decline [28]. As atrial emptying is more dependent on pump function in older age [29], our results indicated that atrial pump function may have greater potential for improvement through SMI enhancement than atrial reservoir function.

The loss of skeletal muscle mass, or sarcopenia, is associated with adverse outcomes across a spectrum of cardiac conditions. Patients with cardiovascular disease often exhibit accelerated muscle loss compared to healthy individuals [30]. Also, reduced muscle mass is associated with increased mortality in heart failure [31]. Further, sarcopenia may be an initial sign of deterioration in patients with heart disease. For example, reduced psoas muscle mass has been identified as an independent predictor of mortality in older adults undergoing transcatheter aortic valve replacement, underscoring the prognostic value of muscle mass in cardiac populations [32]. Conversely, exercise training serves as a nonpharmacological therapeutic modality to restore impaired cardiovascular function, with resistance training being particularly effective in restoring muscle mass and metabolic health [33]. Whether resistance training can improve left atrial function and confer cardioprotective benefits by increasing the SMI in older or sarcopenic adults remains a hypothesis to be tested in future longitudinal or interventional studies.

The statistical mediation effects of skeletal muscle mass varied across sex and age subgroups, as well as among upper- and lower-body muscles. Our data suggested that the indirect association of the SMI with the PA–LA relationship tended to be stronger in women than in men, though this trend should be considered exploratory. This observation aligns with previous reports suggesting that the protective effect of PA against atrial fibrillation is stronger in females [34]. Similarly, a prospective study of 412,413 participants demonstrated that women derived greater benefit from leisure-time PA in reducing all-cause and cardiovascular mortality risk compared with men [35]. These sex-specific differences may be attributable, in part, to variations in muscle fiber characteristics and a higher capillary density per unit of skeletal muscle in females [36]. Likewise, our results suggested that the mediating effect of the SMI tended to be stronger among participants aged ≥60 years compared to those under 60. A hypothesis that warrants future investigation is that age-related muscle loss, together with lower absolute baseline muscle mass in older adults, may make resistance training relatively more effective in this population. This notion is supported by a large meta-analysis indicating that a lower daily step count (6000–8000 steps) is sufficient to reduce mortality risk in adults aged 60 years and older, whereas a higher threshold (8000–10,000 steps) is required for younger adults [37]. These findings indicate that sex and age differences may have implications for exercise prescription, but further research is needed to confirm this potential. In addition, the mediation effect of the USMI tended to be smaller than that of the LSMI in the PA and LA strain relationship. But the USMI and LSMI are not independent mediators; they are derived from the same muscle mass assessment and together approximate the SMI. No formal statistical comparison of the indirect effects of the LSMI and USMI was performed; the difference in their mediation proportions is exploratory. However, the observed difference in their mediating effects warrants future investigation into whether lower-limb muscle training plays a distinct role in rehabilitation settings. For instance, in stroke patients, an observational study demonstrated that lower-limb muscle mass was most strongly associated with the recovery of activities of daily living [38].

The interpretation of this study should be considered in the context of several limitations. First, this study has a cross-sectional design. Consequently, the temporal precedence required for causal inference could not be fully established. Although causal mediation analysis is used to denote the statistical framework [22], this does not imply that we have demonstrated a true causal pathway. Our findings should therefore be interpreted as associations that are consistent with a mediational hypothesis, rather than as definitive evidence of causation. Future studies could employ longitudinal randomized controlled trials to verify causal relationships between variables. Second, subgroup analyses were exploratory in nature and were not powered for formal hypothesis testing. Although we observed numerical differences in the statistical mediation effects across sex and age subgroups, these subgroup analyses were exploratory and were not formally tested for statistical interaction, and the 95% confidence intervals should be considered when interpreting the precision of our estimates. Future studies with adequate power for interaction testing are needed to confirm whether the statistical mediating role of the SMI differs by sex or age. Third, leisure-time PA over the preceding three months was assessed using the IPAQ. Although the IPAQ is one of the most widely used and validated questionnaires for assessing PA in epidemiological research, it is inherently subject to recall bias. Also of note is that bioelectrical impedance analysis for skeletal muscle mass can introduce measurement variability. InBody provides an estimate of skeletal muscle mass and shows that bioelectrical impedance measurements may be influenced by hydration status and other physiological factors. Objective measures of PA and skeletal muscle (e.g., accelerometers, computerized tomography) in future research would strengthen the validity of the findings. Fourth, although we adjusted for a wide range of potential confounders, the possibility of residual or unmeasured confounding could not be ruled out. Factors such as dietary intake, genetic predisposition, occupational PA, or subclinical inflammation were not measured in this study and could have influenced the observed associations. Fifth, the risk of false-positive findings is inherent in our analytical approach. To mitigate this, we applied Benjamini–Hochberg FDR correction to the primary mediation analyses. However, subgroup analyses were exploratory and were not subjected to FDR correction, and the possibility of chance findings cannot be completely excluded. Sixth, detailed characteristics of the participants with missing data on PA, body composition, and echocardiography were not available in our dataset, and thus we could not compare them with the analytic sample. This may introduce selection bias. Exclusion of participants with injuries or physical limitations could reduce the generalizability of our findings to individuals with functional impairments. Finally, in the context of a limited sample, our findings suggest a detectable statistical mediation effect rather than a large explanatory effect. Therefore, all results should be interpreted with appropriate caution, and future studies are needed to determine whether interventions targeting the SMI yield meaningful improvements in LA function.

## 5. Conclusions

In this community-based cross-sectional study, higher levels of leisure-time PA were associated with more favorable LA strain parameters in a Chinese adult population. Skeletal muscle mass (especially quantified by the SMI) statistically accounted for part of these associations. The mediation effects tended to be larger in female participants and those aged ≥60 years. However, given the cross-sectional design, these results should be interpreted as hypothesis-generating. The observed variations in the mediation effect across sex and age subgroups suggested that these factors may deserve consideration in the association between PA and LA function. Future longitudinal or interventional studies are needed to confirm these observational findings and to determine whether strategies aimed at preserving or increasing muscle mass could contribute to maintaining cardiac function in aging populations.

## Figures and Tables

**Table 1:** Baseline characteristics of participants.

Characteristics	Overall	LA Reservoir Strain		*p*-Value
		<40%	≥40%	
	(*n* = 407)	(*n* = 147)	(*n* = 260)	
Age	56.61 ± 11.59	62.38 ± 11.02	53.35 ± 10.6	<0.001
Male sex	177 (43.5%)	68 (46.3%)	109 (41.9%)	0.41
Body mass index (kg/m^2^)	24.85 ± 3.44	23.81 ± 3.24	25.43 ± 3.42	<0.001
Current smoker	87 (21.4%)	33 (22.4%)	54 (20.7%)	0.69
Postmenopausal (female only, n = 230)	148 (64.3%)	63 (79.7%)	85 (56.3%)	<0.001
Hypertension	124 (30.5%)	49 (33.3%)	75 (28.8%)	0.37
Diabetes	71 (17.4%)	33 (22.4%)	38 (14.6%)	0.06
Coronary heart disease	28 (6.9%)	19 (12.9%)	9 (3.5%)	<0.001
Sitting time (h)	4.24 ± 2.71	3.89 ± 2.31	4.44 ± 2.90	0.05
Systolic blood pressure (mmHg)	134.4 ± 19.6	137.3 ± 18.9	132.7 ± 19.8	0.02
Diastolic blood pressure (mmHg)	82.5 ± 10.9	81.1 ± 9.0	83.3 ± 11.8	0.05
Antihypertensive drug	60 (14.7%)	23 (15.6%)	37 (14.2%)	0.70
Antidiabetic drug	36 (8.8%)	18 (12.2%)	18 (6.9%)	0.07
Triglyceride (mmol/L)	1.81 ± 1.22	1.75 ± 1.10	1.84 ± 1.30	0.46
LDL cholesterol (mmol/L)	3.06 ± 0.74	3.01 ± 0.82	3.09 ± 0.70	0.31
Serum creatinine (μmol/L)	66.42 ± 16.00	68.75 ± 15.19	65.11 ± 16.31	0.03
HbA1c (%)	5.69 ± 0.95	5.72 ± 0.93	5.68 ± 0.96	0.68
Echocardiographic variables				
LV end-diastolic volume index (mL/m^2^)	55.54 ± 7.51	54.46 ± 8.69	56.15 ± 6.69	0.03
LV ejection fraction (%)	62.46 ± 2.05	62.40 ± 2.41	62.49 ± 1.81	0.66
LV mass index (g/m^2^)	89.15 ± 25.80	91.50 ± 19.86	87.82 ± 28.56	0.17
E/e’	9.40 ± 1.98	9.64 ± 2.15	9.27 ± 1.87	0.07
LV longitudinal strain (%)	−22.80 ± 1.88	−21.80 ± 2.25	−23.37 ± 1.35	<0.001
LA volume index (mL/m^2^)	23.96 ± 5.70	25.50 ± 7.05	23.08 ± 4.56	<0.001
LA reservoir strain (%)	43.58 ± 10.57	32.01 ± 5.74	50.12 ± 6.15	<0.001
LA conduit strain (%)	−22.87 ± 8.13	−15.94 ± 4.68	−26.79 ± 6.97	<0.001
LA pump strain (%)	−20.71 ± 6.33	−16.07 ± 5.10	−23.33 ± 5.39	<0.001
Physical activity classification				<0.001
≥1500 MET-min/week	23 (5.7%)	2 (1.4%)	21 (8.1%)	—
1000–1499 MET-min/week	92 (22.6%)	14 (9.5%)	78 (30.0%)	—
500–999 MET-min/week	154 (37.8%)	48 (32.7%)	106 (40.8%)	—
<500 MET-min/week	121 (29.7%)	67 (45.6%)	54 (20.8%)	—
totally sedentary	17 (4.2%)	16 (10.9%)	1 (0.4%)	—
Fat-free muscle index (kg/m^2^)	16.62 ± 1.83	16.05 ± 1.78	16.94 ± 1.78	<0.001
Skeletal muscle index (kg/m^2^)	6.71 ± 0.93	6.37 ± 0.89	6.90 ± 0.90	<0.001
Skeletal muscle index of upper limbs (kg/m^2^)	1.72 ± 0.33	1.62 ± 0.33	1.77 ± 0.33	<0.001
Skeletal muscle index of lower limbs (kg/m^2^)	4.99 ± 0.63	4.74 ± 0.60	5.13 ± 0.60	<0.001
Percent body fat (kg)	32.55 ± 6.78	32.03 ± 6.98	32.85 ± 6.66	0.24
Grip strength (kg)	23.59 ± 8.48	21.89 ± 8.52	24.55 ± 8.31	0.002

Values are mean ± standard deviation for normally distributed continuous variables, *n* (%) for categorical variables. LA, left atrial; LV, left ventricle; LDL, low-density lipoprotein.

**Table 2:** Total, direct and indirect effects of PA on LA function mediated by SMI.

LA Strain	Group of Participants	Total Effect	Average Direct Effect	Average Causal Mediation Effect	Proportion Mediated	*p*-Value for Mediation Effect
		β (95% CI)	β (95% CI)	β (95% CI)	β (95% CI)	
LA reservoir strain	All (*n* = 407)	2.91 (1.99, 3.88)	2.31 (1.39, 3.39)	0.60 (0.27, 1.04)	0.21 (0.09, 0.38)	<0.001
LA reservoir strain	Male (*n* = 177)	2.78 (1.32, 4.28)	2.41 (0.78, 3.90)	0.37 (0.02, 0.94)	0.13 (0.01, 0.48)	0.040
LA reservoir strain	Female (*n* = 230)	3.04 (1.70, 4.20)	2.27 (0.83, 3.54)	0.77 (0.31, 1.31)	0.25 (0.10, 0.55)	<0.001
LA reservoir strain	≥60 y (*n* = 156)	2.81 (1.24, 4.29)	1.89 (0.15, 3.36)	0.92 (0.33, 1.71)	0.33 (0.11, 0.86)	0.006
LA reservoir strain	<60 y (*n* = 251)	2.95 (1.68, 4.11)	2.46 (1.15, 3.69)	0.49 (0.13, 0.91)	0.17 (0.04, 0.38)	0.006
LA pump strain	All (*n* = 407)	−1.67 (−2.24, −1.07)	−1.07 (−1.67, −0.46)	−0.60 (−0.86, −0.36)	0.36 (0.20, 0.59)	<0.001
LA pump strain	Male (*n* = 177)	−1.38 (−2.28, −0.35)	−1.02 (−1.87, 0.05)	−0.36 (−0.79, −0.06)	0.26 (0.03, 0.99)	0.036
LA pump strain	Female (*n* = 230)	−1.98 (−2.69, −1.22)	−1.22 (−2.01, −0.43)	−0.75 (−1.16, −0.39)	0.38 (0.21, 0.65)	<0.001
LA pump strain	≥60 y (*n* = 156)	−2.17 (−3.19, −1.07)	−1.39 (−2.46, −0.25)	−0.78 (−1.39, −0.34)	0.36 (0.14, 0.78)	<0.001
LA pump strain	<60 y (*n* = 251)	−1.41 (−2.06, −0.65)	−0.92 (−1.65, −0.13)	−0.49 (−0.78, −0.24)	0.35 (0.16, 0.81)	0.002

LA, left atrial; PA, physical activity; SMI, skeletal muscle mass index.

## Data Availability

The datasets used and analyzed in this current study are available from the corresponding authors on reasonable request.

## Supplementary Materials

The following supporting information can be downloaded at https://www.mdpi.com/article/10.3390/jcm15197744/s1, Figure S1: Flowchart of the inclusion process; Figure S2: Correlations between dose of physical activity, indices of skeletal muscle mass and left atrial strain; Table S1: The association between physical activity and left atrial strain in all the participants and across subgroups; Table S2: The statistical mediation effect of fat free mass index (FFMI), upper limbs skeletal muscle mass index (USMI) and lower limbs skeletal muscle mass index (LSMI).

## Abbreviations

The following abbreviations are used in this manuscript:

FFMI	Fat-free mass index
HLPC	Healthy Lifestyle Promotion Cohort
IPAQ	International Physical Activity Questionnaire
LA	Left atrial
LSMI	Skeletal muscle mass index of lower limbs
LV	Left ventricular
LVEF	Left ventricular ejection fraction
LVGLS	LV global longitudinal strain
MET	Metabolic equivalent of task
PA	Physical activity
SMI	Skeletal muscle mass index
USMI	Skeletal muscle mass index of upper limbs
