# Supplementary material for: Skeletal Muscle Mass as a Potential Mediator of the Physical Activity–Left Atrial Function Relationship: A Community-Based Cross-Sectional Study
Source: J Clin Med. 2026 Oct 8;15(19):7744. doi: 10.3390/jcm15197744 (PMC13666086; doi:10.3390/jcm15197744)
Supplement: Supplementary file 1 [file jcm-15-07744-s001.zip › jcm-4492192-supplementary.pdf]

Supplementary Fig. S1 Flowchart of the inclusion process.

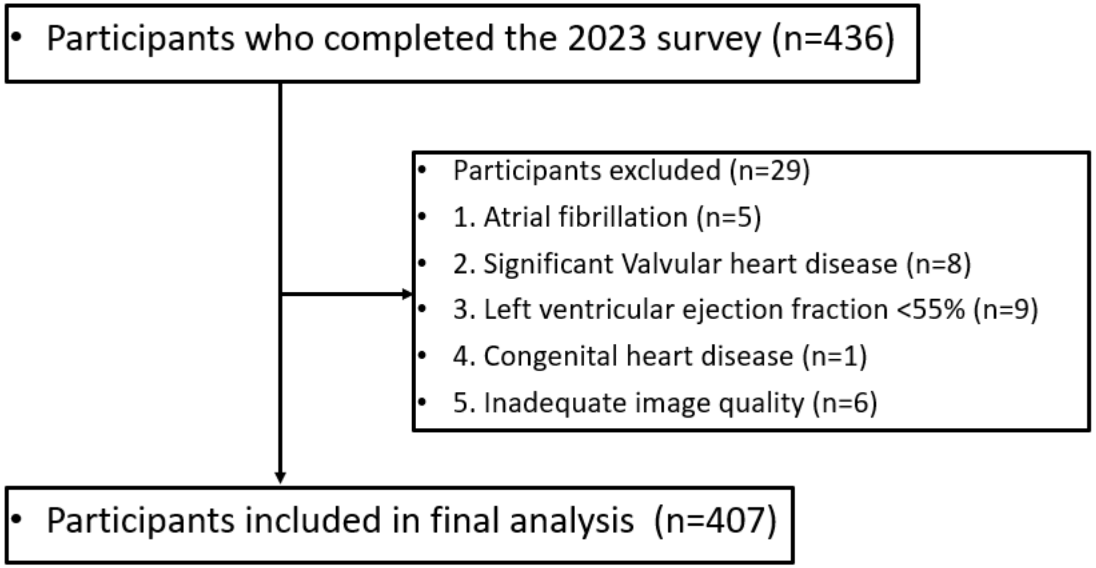

Supplementary Fig. S2 Correlations between dose of physical activity, indices of skeletal muscle mass and left atrial strain.

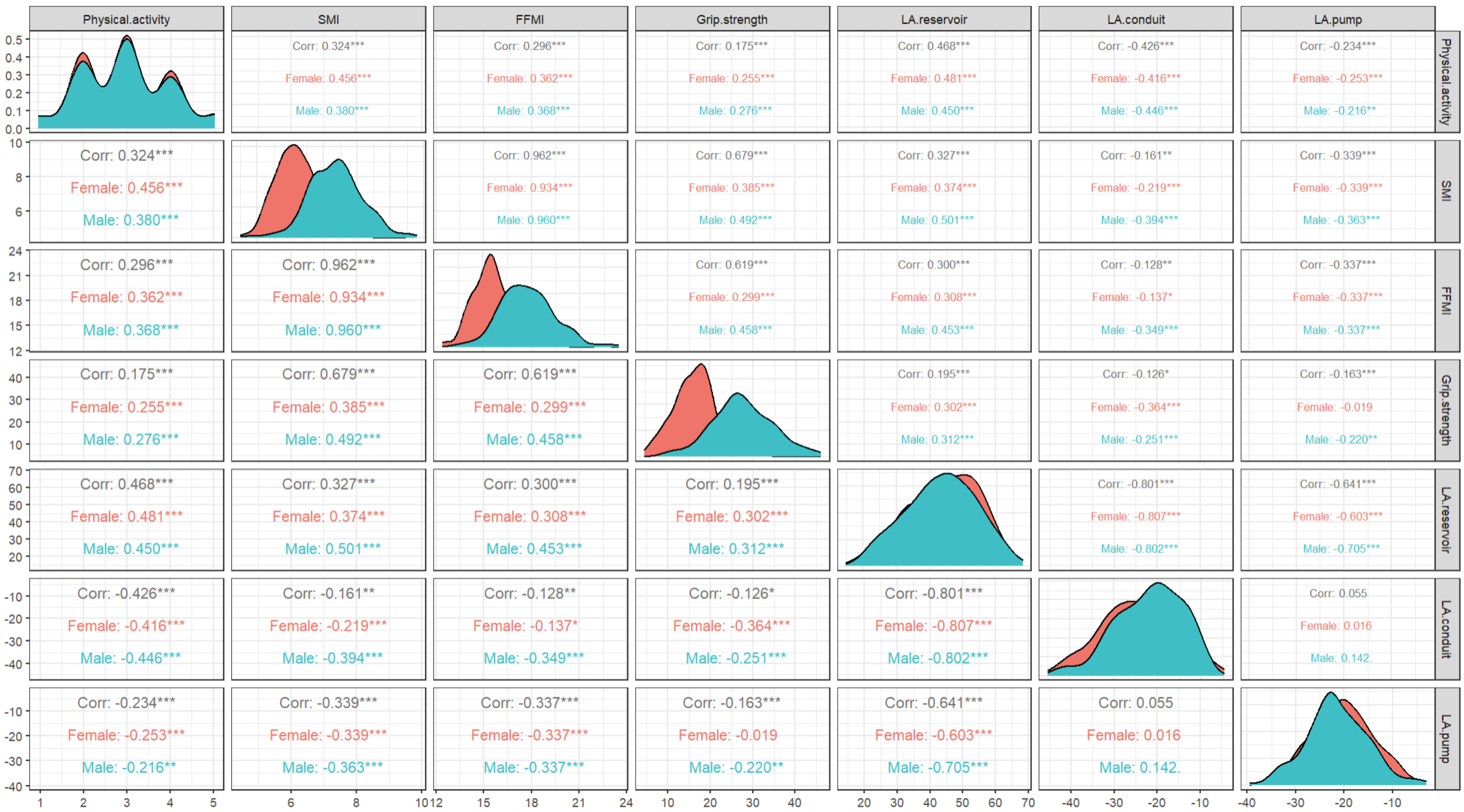

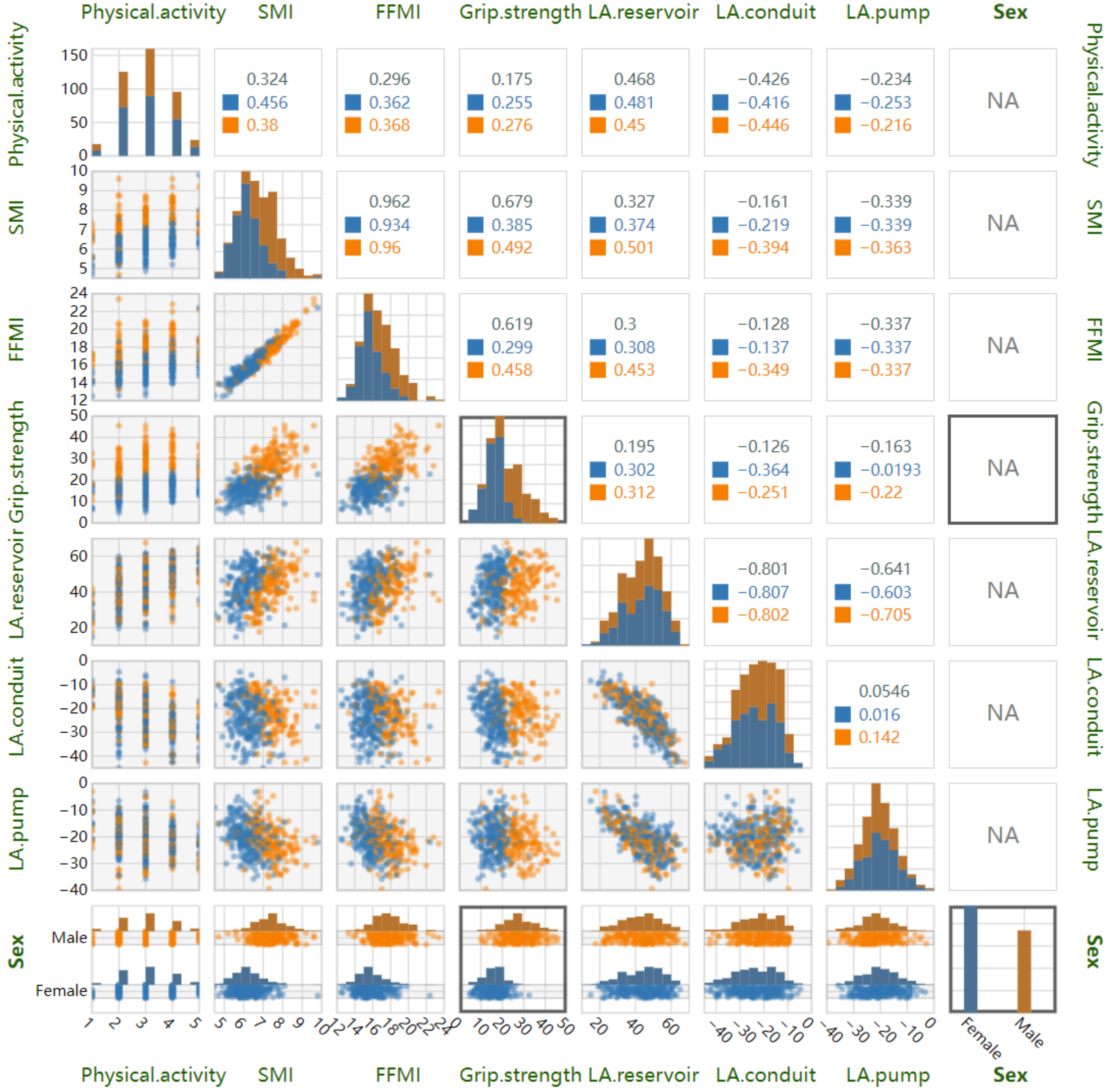
Physical.activity
SMI
FFMI
Grip.strength
LA.reservoir
LA.conduit
LA.pump
Sex
0.324
0.456
0.38
0.296
0.362
0.368
0.175
0.255
0.276
0.468
0.481
0.45
−0.426
−0.416
−0.446
−0.234
−0.253
−0.216
NA
0.962
0.934
0.96
0.679
0.385
0.492
0.327
0.374
0.501
−0.161
−0.219
−0.394
−0.339
−0.339
−0.363
NA
0.619
0.299
0.458
0.3
0.308
0.453
−0.128
−0.137
−0.349
−0.337
−0.337
−0.337
NA
0.195
0.302
0.312
−0.126
−0.364
−0.251
−0.163
−0.0193
−0.22
NA
−0.801
−0.807
−0.802
−0.641
−0.603
−0.705
NA
0.0546
0.016
0.142
NA
NA
Male
Female

Supplementary Table S1. The association between physical activity and left atrial strain in all the participants and across subgroups. Model 1 included adjustment for age, sex, left ventricular ejection fraction, the comorbidity of hypertension, diabetes and coronary heart disease. Model 2 added left ventricular longitudinal strain, and Model 3 furtherly added the use of antihypertensive drugs and antidiabetic drugs.

**Outcome**	**Participants analyzed**	**Model 1** β (95%CI)	**Model 2** β (95%CI)	**Model 3** β (95%CI)
Left atrial reservoir strain	All (n=407)	3.594 (2.591,4.596)	2.900 (2.004,3.795)	2.907 (2.010,3.805)
	Male (n=177)	3.288 (1.783,4.793)	2.773 (1.420,4.127)	2.777 (1.411,4.142)
	Female (n=230)	3.881 (2.531,5.231)	3.042 (1.842,4.242)	3.037 (1.830,4.243)
	≥60 (n=156)	3.787 (2.093,5.482)	2.862 (1.368,4.356)	2.812 (1.307,4.317)
	< 60 (n=251)	3.412 (2.118,4.707)	2.941 (1.770,4.111)	2.945 (1.778,4.112)
Left atrial pump strain	All (n=407)	-2.011 (-2.696,-1.326)	-1.667 (-2.319,-1.016)	-1.674 (-2.328,-1.021)
	Male (n=177)	-1.637 (-2.705,-0.569)	-1.353 (-2.362,-0.344)	-1.375 (-2.391,-0.359)
	Female (n=230)	-2.351 (-3.255,-1.446)	-1.965 (-2.831,-1.098)	-1.976 (-2.847,-1.105)
	≥60 (n=156)	-2.749 (-4.008,-1.491)	-2.202 (-3.377,-1.026)	-2.169 (-3.353,-0.985)
	< 60 (n=251)	-1.612 (-2.423,-0.800)	-1.406 (-2.184,-0.628)	-1.409 (-2.190,-0.629)
Left atrial conduit strain	All (n=407)	-1.583 (-2.254,-0.911)	-1.232 (-1.868,-0.597)	-1.233 (-1.870,-0.596)
	Male (n=177)	-1.652 (-2.632,-0.671)	-1.421 (-2.361,-0.481)	-1.402 (-2.350,-0.455)
	Female (n=230)	-1.530 (-2.461,-0.600)	-1.077 (-1.952,-0.203)	-1.061 (-1.937,-0.185)
	≥60 (n=156)	-1.038 (-1.984,-0.093)	-0.66 (-1.556,0.236)	-0.643 (-1.547,0.261)
	< 60 (n=251)	-1.801 (-2.720,-0.882)	-1.535 (-2.403,-0.668)	-1.536 (-2.400,-0.672)

Supplementary Table S2. The statistical mediation effect of fat free mass index (FFMI), upper limbs skeletal muscle mass index (USMI) and lower limbs skeletal muscle mass index (LSMI).

		Mediator	Average Causal Mediation Effect	Average Direct Effect	Total effect	Mediation effect
LA reservoir strain	All (n=407)	FFMI	0.47 (0.18, 0.81) p<0.001	2.44 (1.35, 3.39) p<0.001	2.91(1.90, 3.79) p<0.001	0.16 (0.06, 0.32) p<0.001
	Male (n=177)	FFMI	0.31 (-0.01, 0.85) p=0.076	2.47 (0.81, 3.93) p<0.001	2.78 (1.32, 4.08) p<0.001	0.11 (-0.01,0.43) P=0.076
	Female (n=230)	FFMI	0.65 (0.25, 1.11) p=0.002	2.38 (1.05, 3.75) p<0.001	3.04 (1.73, 4.34) p<0.001	0.22 (0.08, 0.44) p=0.002
	≥60 (n=156)	FFMI	0.71 (0.24, 1.35) p=0.002	2.10 (0.44, 3.60) p=0.012	2.81 (1.19, 4.28) p<0.001	0.25 (0.09, 0.69) p=0.002
	< 60 (n=251)	FFMI	0.41 (0.04, 0.84) p=0.032	2.54 (1.22, 3.80) p<0.001	2.95 (1.70, 4.14) p<0.001	0.14 (0.01, 0.35) p=0.032
LA pump strain	All (n=407)	FFMI	-0.50 (-0.76, -0.27) p<0.001	-1.18 (-1.79, -0.59) P=0.002	-1.67 (-2.24, -1.13) p<0.001	0.30 (0.15, 0.51) p<0.001
	Male (n=177)	FFMI	-0.32 (-0.71, -0.05) p=0.016	-1.05 (-2.01, -0.02) p=0.050	-1.38 (-2.30, -0.45) p=0.006	0.23 (0.04, 0.95) p=0.022
	Female (n=230)	FFMI	-0.64 (-0.99, -0.32) p<0.001	-1.33 (-2.07, -0.59) p<0.001	-1.98 (-2.68, -1.23) p<0.001	0.32 (0.16, 0.56) p<0.001
	≥60 (n=156)	FFMI	-0.65 (-1.18, -0.25) p<0.001	-1.52 (-2.63, -0.39) p=0.010	-2.17 (-3.28, -1.06) p<0.001	0.30 (0.11, 0.68) p<0.001
	< 60 (n=251)	FFMI	-0.43 (-0.77, -0.18) p<0.001	-0.98 (-1.71, -0.16) p=0.012	-1.41 (-2.09, -0.63) p<0.001	0.30 (0.12, 0.79) p<0.001
LA reservoir strain	All (n=407)	USMI	0.42 (0.14, 0.76) p<0.001	2.49 (1.53, 3.44) p<0.001	2.91 (2.00, 3.84) p<0.001	0.14 (0.04, 0.29) p<0.001
	Male (n=177)	USMI	0.29 (-0.08, 0.80) p=0.114	2.48 (0.77, 4.04) p=0.006	2.78 (1.23, 4.18) p<0.001	0.11 (-0.02, 0.40) p=0.114
	Female (n=230)	USMI	0.55 (0.17, 1.02) p=0.002	2.49 (1.07, 3.81) p<0.001	3.04 (1.70, 4.29) p<0.001	0.18 (0.06, 0.42) p=0.002
	≥60 (n=156)	USMI	0.66 (0.17, 1.35) p=0.008	2.15 (0.39, 3.70) p=0.010	2.81 (1.10, 4.39) p=0.004	0.23 (0.06, 0.64) p=0.012

	< 60 (n=251)	USMI	0.33 (0.03, 0.76)<br>p=0.032	2.61 (1.21, 3.96)<br>p<0.001	2.95 (1.62, 4.17)<br>p<0.001	0.11 (0.01, 0.31)<br>p=0.032
LA pump strain	All (n=407)	USMI	-0.50 (-0.73, -0.27)<br>p<0.001	-1.18 (-1.80, -0.56)<br>p=0.002	-1.67 (-2.25, -1.08)<br>p<0.001	0.30 (0.16, 0.53)<br>p<0.001
	Male (n=177)	USMI	-0.35 (-0.73, -0.08)<br>p<0.001	-1.03 (-1.95, 0.08)<br>p=0.066	-1.38 (-2.26, -0.32)<br>p=0.008	0.25 (0.05, 1.05)<br>p=0.008
	Female (n=230)	USMI	-0.60 (-0.97, -0.29)<br>p<0.001	-1.38 (-2.15, -0.54)<br>p<0.001	-1.98(-2.69, -1.21)<br>p<0.001	0.30 (0.14, 0.58)<br>p<0.001
	≥60 (n=156)	USMI	-0.63 (-1.15, -0.22)<br>p<0.001	-1.53 (-2.64, -0.39)<br>p=0.010	-2.17 (-3.13, -1.13)<br>p<0.001	0.29 (0.10, 0.69)<br>p<0.001
	< 60 (n=251)	USMI	-0.41(-0.73, -0.15)<br>p<0.001	-1.00 (-1.70, -0.26)<br>p=0.010	-1.41 (-2.08, -0.72)<br>p<0.001	0.29 (0.11, 0.71)<br>p<0.001
LA reservoir strain	All (n=407)	LSMI	0.60 (0.31, 1.00)<br>p<0.001	2.31 (1.29, 3.28)<br>p<0.001	2.91 (1.99, 3.82)<br>p<0.001	0.21 (0.10, 0.38)<br>p<0.001
	Male (n=177)	LSMI	0.33 (0.01, 0.88)<br>p=0.044	2.44 (0.84, 3.98)<br>p=0.002	2.78 (1.32, 4.24)<br>p<0.001	0.12 (0.01, 0.42)<br>p=0.044
	Female (n=230)	LSMI	0.73 (0.25, 1.23)<br>p=0.002	2.30 (0.90, 3.61)<br>p=0.002	3.04 (1.70, 4.28)<br>p<0.001	0.24 (0.07, 0.54)<br>p=0.002
	≥60 (n=156)	LSMI	0.89 (0.32, 1.74)<br>p<0.001	1.92 (0.24, 3.47)<br>p=0.024	2.81 (1.09, 4.36)<br>p=0.002	0.32 (0.11, 0.82)<br>p=0.002
	< 60（n=251）	LSMI	0.49 (0.13, 0.92)<br>p<0.001	2.45 (1.11, 3.80)<br>p<0.001	2.95 (1.69, 4.16)<br>p<0.001	0.18 (0.04, 0.38)<br>p<0.001
LA pump strain	All (n=407)	LSMI	-0.57 (-0.83, -0.34)<br>p<0.001	-1.11(-1.69, -0.50)<br>p<0.001	-1.67 (-2.26, -1.07)<br>p<0.001	0.34 (0.19, 0.58)<br>p<0.001
	Male (n=177)	LSMI	-0.31 (-0.69, -0.01)<br>p=0.040	-1.07 (-1.93, -0.07)<br>p=0.042	-1.37 (-2.23, -0.41)<br>p=0.008	0.22 (0.01, 0.85)<br>p=0.048
	Female (n=230)	LSMI	-0.69 (-1.09, -0.33)<br>p=0.002	-1.29 (-2.01, -0.50)<br>p<0.001	-1.98 (-2.68, -1.28)<br>p<0.001	0.35 (0.17, 0.65)<br>p=0.002
	≥60 (n=156)	LSMI	-0.73 (-1.35, -0.29)<br>p<0.001	-1.44 (-2.47, -0.28)<br>p=0.012	-2.17 (-3.20, -1.09)<br>p=0.002	0.33 (0.13, 0.75)<br>p=0.002
	< 60 (n=251)	LSMI	-0.46 (-0.77, -0.21)<br>p<0.001	-0.95 (-1.68, -0.21)<br>p=0.014	-1.41 (-2.09, -0.69)<br>p<0.001	0.33 (0.14, 0.76)<br>p<0.001
